# Adiponectin and phase angle in the assessment of sarcopenia in Crohn’s disease: beyond muscle mass

**DOI:** 10.3389/fnut.2026.1753532

**Published:** 2026-04-23

**Authors:** Ersilia Nigro, Maurizio Marra, Francesca De Santis, Rosa Sammarco, Camilla Leo, Olga Maria Nardone, Giulio Calabrese, Alessia Dalila Guarino, Fabiana Castiglione, Aurora Daniele, Fabrizio Pasanisi

**Affiliations:** 1 CEINGE-Biotecnologie Avanzate Scarl “Franco Salvatore”, Napoli, Italy; 2 Dipartimento di Scienze e Tecnologie Ambientali, Biologiche, Farmaceutiche, Università della Campania “Luigi Vanvitelli”, Caserta, Italy; 3 UOC Medicina Interna e Nutrizione, Clinica Department of Clinical Medicine and Surgery, University of Naples “Federico II”, Naples, Italy; 4 Gastroenterology Unit, Department of Clinical Medicine and Surgery, University of Naples “Federico II”, Naples, Italy; 5 Gastroenterology, Department of Public Health, University of Naples “Federico II”, Napoli, Italy; 6 Dipartimento di Medicina Molecolare e Biotecnologie Mediche, Università di Napoli “Federico II”, Napoli, Italy

**Keywords:** adiponectin, Crohn’s disease, inflammation, phase angle, sarcopenia

## Abstract

**Introduction:**

Sarcopenia is a common and clinically relevant complication of Crohn’s disease (CD), reflecting the cross-link among chronic inflammation, malnutrition, and metabolic dysregulation. Adiponectin, an anti-inflammatory adipokine, and phase angle, a marker of cellular integrity, have emerged as potential indicators of muscle health. This study aimed to evaluate the role of anthropometric and inflammation parameters in 150 CD patients.

**Methods:**

Anthropometric and bioimpedance parameters including handgrip strength and PhA were collected to define sarcopenia according to EWGSOP2. Additionally, the levels of adiponectin and inflammatory cytokines (IL-6, IL-1β, TNF-*α*, CRP) were tested by ELISA. Adiponectin oligomeric status was assessed by Western Blotting.

**Results:**

Sarcopenia was identified in 8% and pre-sarcopenia in 71% of patients. Interestingly, sarcopenic and pre-sarcopenic patients were significantly younger and had lower BMI, fat-free mass, and skeletal mass compared to non-sarcopenic individuals (*p* < 0.05). Phase angle values were also statistically lower in sarcopenic subjects (5.06 ± 0.67°). Adiponectin levels were significantly reduced in sarcopenic (14.0 ± 5.2 μg/mL) compared to pre-sarcopenic and non-sarcopenic patients (20.1 ± 4.5, 20.2 ± 3.6 μg/mL, respectively *p* < 0.05). The ROC analysis identified both adiponectin and PhA as accurate predictors of sarcopenia risk in CD. Analysis of adiponectin oligomers evidenced a significant down regulation of HMW and MMW isoforms in sarcopenic vs. pre-sarcopenic and non-sarcopenic patients.

**Discussion:**

Our findings support the pivotal role of metabolic and nutritional disorders in muscle loss in CD patients and indicate adiponectin and phase angle as complementary biomarkers of sarcopenia. Furthermore, our results, highlight the interplay among inflammation, adipose tissue dysfunction, and muscle wasting in Crohn’s disease.

## 1 Introduction

Crohn’s disease (CD), one of the main forms of inflammatory bowel disease (IBD), is a chronic, relapsing inflammatory disease affecting the gastrointestinal tract (1). Patients with CD are characterized by a clinical phenotype resulting from diverse and complex interactions between genetic, environmental, and immune factors (2). Furthermore, CD condition is increasingly recognized as systemic, affecting not only the intestine but also the metabolism and body composition of the entire organism (3). Indeed, among the extraintestinal consequences, there is a significant aggravating factor, the sarcopenia, i.e., a progressive loss of skeletal muscle mass and strength that affects up to 40–50% of patients with IBD (4). Sarcopenia in CD is mainly due to chronic inflammation, malnutrition, reduced physical activity, and alterations in the gut-muscle axis. In this scenario, adipose tissue plays a key role in CD because it, functioning as an active endocrine organ, releases several adipokines implicated in the modulation of systemic metabolism and inflammation (5, 6). Chronic alterations in adipose tissue can influence muscle metabolism, promote catabolism and exacerbate muscle loss due to systemic inflammation, insulin resistance, and altered hormonal signaling (7). Inflamed adipose tissue releases proinflammatory cytokines (e.g., TNF-*α*, IL-6) and adipokines that contribute to influencing muscle metabolism, promoting catabolism and exacerbating muscle loss. Conversely, adiponectin appears to exert anti-inflammatory and insulin-sensitizing effects, although its precise role in CD remains controversial. Adiponectin plays a particularly important role in metabolic and chronic diseases (8); it circulates in three main isoforms with different molecular weights: low molecular weight (LMW), medium molecular weight (MMW), and high molecular weight (HMW). The LMW form is a trimer (~90 kDa), the MMW form is a hexamer (~180 kDa), and the HMW form is a large multimer of 12–36 units (over 400 kDa). The HMW isoform is generally considered the most biologically active (8). Interestingly, adiponectin generally exhibits protective metabolic effects, i.e., enhances fatty acid oxidation, improves insulin sensitivity, and exerts anti-inflammatory and antioxidant effects (9). In chronic conditions, adiponectin has been shown to protect against muscle wasting. Adiponectin also stimulates satellite cell activation, myoblast differentiation, and mitochondrial biogenesis, contributing to muscle regeneration and maintenance (10). In the light of this evidence, the study aimed to evaluate the role of anthropometric and inflammation parameters, as predictors of sarcopenia in CD patients. In addition, Adiponectin and its oligomers serum expression was investigated.

## 2 Methods

### 2.1 Design of the Study and Patients Population

This is a retrospective analysis that includes a cohort of 150 consecutive adult CD outpatients recruited from the Gastroenterology Unit, Department of Clinical Medicine and Surgery, University of “Federico II,” Naples (Italy). As previously described (11, 12), the inclusion criteria were a diagnosis of CD and an age range of 18 to 65 years. Exclusion criteria included the use of corticosteroids in the last 3 months, a history of acute or chronic liver or kidney disease, current enteral or parenteral nutrition, presence of fistulae, ileostomy or colostomy, extensive small bowel resections (residual small bowel <2 m), pregnancy or lactation and unstable body weight in the last month. The study adhered to the Declaration of Helsinki and was approved by the Federico II Ethical Committee (No. 102/16) and registered on clinicaltrials.gov as NCT03054935 (November 2016). All participants provided written informed consent before enrolment.

### 2.2 Anthropometric parameters and muscle strength measurement

The analysis of anthropometric and body composition parameters included measurements of weight and height, calculation of BMI, and determination of percentage of fat mass (FM) and free fat mass (FFM). For each subject, height and weight were obtained using standard techniques; BMI was calculated as body weight (kg)/height^2^ (m^2^). BIA was performed at 50 kHz (Human In Plus II, DS Medica, Milan, Italy) with a constant room temperature of 22–25 °C. Measurements were carried out on the nondominant side of the body, in the postabsorptive state, after voiding and with the subject in the supine position for 20 min. The BIA was conducted according to the Organ method (13). Finally, FFM and FM were estimated by using the predictive equations developed by Sun (14). Skeletal mass (SM) was calculated using the Sergi BIA equation (15). Additionally, handgrip strength (HGS) was used to assess the force generated by the hand muscles using a dynamometer (JAMAR, Roylan, UK). Participants stood with their arms extended parallel to the trunk and gripped the dynamometer, applying maximum strength with each hand without support. Measurements were taken three times on each side (dominant and nondominant hand) with a 1-min rest between attempts to prevent fatigue (16). The average value was then recorded in kilograms (kg).

### 2.3 Sarcopenia criteria

Sarcopenia was diagnosed according to the EWSGOP2 criteria (17). This new revised consensus suggests that the first step to assess sarcopenia is the presence of dynapenia evaluated with the HGS method (recorded in kilograms). The diagnosis of sarcopenia is confirmed by reduced SM in addition to dynapenia and/or low physical performance. According to the cutoff points of Dodds et al. (18), dynapenia was diagnosed if HGS < 16 kg for females and HGS < 27 for males. Low SM was defined using the Studenski cutoffs: <15 kg for females and ASM < 20 kg for males or Gould cutoffs for SMI (SM/height^2^): <5.5 kg/m^2^ for females and SMI < 7.0 kg/m^2^ for males. In the light of these criteria, we divided our CD population in: (a) sarcopenic patients: those with dynapenia and reduced SM or SMI; (b) pre-sarcopenic patients: those with dynapenia or reduced SM and/or SMI; (c) non sarcopenic patients, those any of the above-mentioned alterations.

### 2.4 Inflammatory parameters

Blood samples were collected to assess various biomarkers related to nutritional status, including C-reactive protein (CRP) (mg/L), fibrinogen (mg/dL), and lymphocytes (19^9^/L). IL-6, IL-1β and TNF-*α* serum concentrations were evaluated using a commercially available enzyme-linked immunosorbent assay (ELISA) kit (BioLegend, San Diego, CA, United States).

### 2.5 Determination of adiponectin and its oligomerization state

Total serum adiponectin concentration was measured by enzyme-linked immunosorbent assay (ELISA). Adiponectin levels were measured using an ELISA developed with a previously characterized in-house polyclonal antibody. Utilizing a polyclonal antibody, in house produced, versus a human adiponectin sequence region (H2N-ETTTQGPGVLLPLPKG-COOH) as previously reported (19). The specificity of this antibody has been previously validated by SDS–PAGE followed by Western blot analysis and protein identification by nanoLC–MS/MS after in-gel trypsin digestion, confirming the immunoreactive band as adiponectin. The analytical performance and validation of this antibody-based assay have been described in detail in our previous study (obesity|VOLUME 16 NUMBER 8|AUGUST 2008 daniele).

Adiponectin oligomerization state was analyzed by western blotting. Briefly, serum samples were quantified for total proteins by Bradford’s method (Bio-Rad, Hercules, CA, United States); 5 μg of total proteins were treated with 1 × Laemmli buffer, heated at 95 °C for 5 min and loaded under non-reducing conditions on 10% SDS-PAGE gel and transferred as previously described (20). The blots were scanned by using ChemiDoc MP imaging system (Bio-Rad, CA, United States) and analyzed by densitometry with ImageJ software^1^. A representative sample of 10 non-sarcopenic, 10 pre-sarcopenic, 10 sarcopenic patients was analyzed and tested two times.

### 2.6 Statistical analysis

Statistical analysis was conducted using SPSS ver. 30.0 (IBM Corporation, Inc., Chicago, IL, United States). Data are presented as the mean ± standard deviation [SD], unless otherwise specified. The Kolmogorove Smirnov Test and the Shapiro Wilk Test were used as tests of normality to examine.

ANOVA or the Mann–Whitney test with post-hoc comparisons were used as appropriate to assess differences among groups. A *p*-value <0.05 was considered significant. Receiver-operating characteristic (ROC) curves were used to identify the parameter that was most associated with the presence of Sarcopenia.

## 3 Results

### 3.1 Anthropometric parameters of CD patients

The analysis of anthropometric parameters of CD patients is shown in Table 1. Weight, height, FFM, SM and SMI were higher in males than females whereas age and FAT % were higher in females. As expected, qualitative parameters (PhA and HGS) were higher in males than females. We found no significative differences between sex for BMI and FAT (kg).

**Table 1:** Anthropometric characteristics in 150 patients with Crohn disease.

Parameters	Measurement unit	Males (*n* = 87)	Females (*n* = 63)	All (*n* = 150)
Age	years	39.0±13.8*	41.6±15.3	40.1±14.5
Weight	kg	69.8±10.5^*^	59.3±10	65.4±11.5
Stature	cm	172±6^*^	159±6	167±9
BMI	kg/m^2^	23.5±3.2	23.3±3.5	23.4±3.3
FFM	kg	57.0±6.0^*^	45.3±4.8	52.1±8.0
FM	kg	12.8±6.4	14.0±6.1	13.3±6.3
FM	%	17.6±6.8^*^	22.7±7.1	19.7±7.4
PhA	degrees	6.49±0.90^*^	5.52±0.58	6.08±0.91
SM	kg	21.3±2.3^*^	15.1±1.8	18.7±3.7
SMI	kg/m^2^	7.17±0.70^*^	5.96±0.53	6.66±0.87
HGS	kg	38.2±8.8^*^	21.3±5.4	31.1±11.3

^*^p < 0.05.

### 3.2 Sarcopenia stratification

Patients were classified as into three groups based on the presence of sarcopenia state or pre-sarcopenia state (Tables 2, 3) after verified that the therapies of the patients had no influence on the sarcopenic status. To analyze whether the sarcopenic state might be related to the severity of the CD, considering the Harvey-Bradshaw index (HBI); 3 severity rates for HBI were considered: 0: <5; 1: >6, & <7; 2: >8 & <16; 3: >16. Firstly, we stratified the patients within the 3 groups of our population study (sarcopenic, pre-sarcopenic, non-sarcopenic) according to HBI value, finding that the distribution of HBI is similar in all 3 groups (Supplementary Figure S1A). Then, we performed a ROC curve analysis considering HDI and found that the severity is not a predictor of the sarcopenic state in our CD population (AUC: 0.474) (Supplementary Figure S1B).

**Table 2:** Anthropometric characteristics, in 150 patients with Crohn disease sub-divided on the basis of sarcopenia status.

Parameters	Meauserement unit	Non-sarcopenic (*n* = 32)	Pre-sarcopenic (*n* = 106)	Sarcopenic (*n* = 12)
Age	years	48.6±15.1^*^	38.2±13.7^*^	33.8±11.2
Weight	kg	72.7±10.4^^^	64.3±10.5^^^	55.4±12.2^^^
Stature	cm	163±8	168±9	165±9
BMI	kg/m^2^	27.1±2.2^^^	22.7±2.7^^^	20.1±3.1^^^
FFM	kg	54.7±8.9^*^	51.9±7.5^*^	46.2±7.4
FM	kg	17.9±5.6^^^	12.3±5.8^^^	9.2±5.9^^^
FM	%	24.7±6.5^^^	18.7±6.9^^^	15.5±7.5^^^
SM	kg	19.3±4.1^*^	18.8±3.4^*^	15.9±3.4
SMI	kg/m^2^	7.15±0.90	6.61±0.77	5.74±0.75
PhA	degrees	5.99±0.97^*^	6.22±0.85^*^	5.06±0.67
HGS	kg	31.1±11.1^*^	32.3±11.2^*^	20.4±5.3

^*^p < 0.05 vs. sarcopenic, ^§^*p* < 0.05 vs. pre-sarcopenic, ^^^*p* < 0.05 between groups. BMI, body mass index; FFM, Fat Free Mass; FM, Fat Mass; SM, Skeletal Mass; SMI, skeletal Mass Index; PhA, phase angle; HGS, handgrip strength.

**Table 3:** Adiponectin and inflammatory markers in 150 patients with Crohn disease.

Parameters	Non-sarcopenic (*n* = 32)	Pre-sarcopenic (*n* = 106)	Sarcopenic (*n* = 12)
Adiponectin	20.1±4.5^*^	20.2±3.6^§^	14.0±5.2
IL_1_beta	7.1±11.4	5.4±9.4	10.5±20.3
IL_6	70±110^*^	65±117	113±136
TNF-alfa	11.1±5.8	10.9±6.6	11.6±5.1
Lymphocytes	1,898±521	1,944±2,196	1,757±829
PCR	9.2±19	7.6±12	15.7±21

^*^p < 0.05 vs. sarcopenic, ^§^*p* < 0.05 vs. pre-sarcopenic.

Sarcopenic and pre-sarcopenic patients were significantly younger, with lower BMI, FFM, skeletal mass, and PhA values than non-sarcopenic counterparts (*p* < 0.05). HGS was markedly reduced only in sarcopenic patients (*p* < 0.001).

Interestingly, we found that pre-sarcopenic and sarcopenic patients were younger than non-sarcopenic; BMI was statistically lower in pre-sarcopenic and sarcopenic patients as also the FFM, PA and SM. HGS values were statistically lower in sarcopenic, but not in pre-sarcopenic compared to non-sarcopenic patients (Table 2).

### 3.3 Inflammatory markers parameters of CD patients subdivided on the basis of sarcopenia status

Next, we analyzed some inflammatory markers; although not significant, we found an increase in PCR and Il-1 beta values in sarcopenic subjects (Table 3). Adiponectin resulted statistically lower in sarcopenic but not in pre-sarcopenic compared to non-sarcopenic patients (Table 3), while IL-6 resulted statistically higher in sarcopenic vs. non-sarcopenic patients.

**Figure 1:**
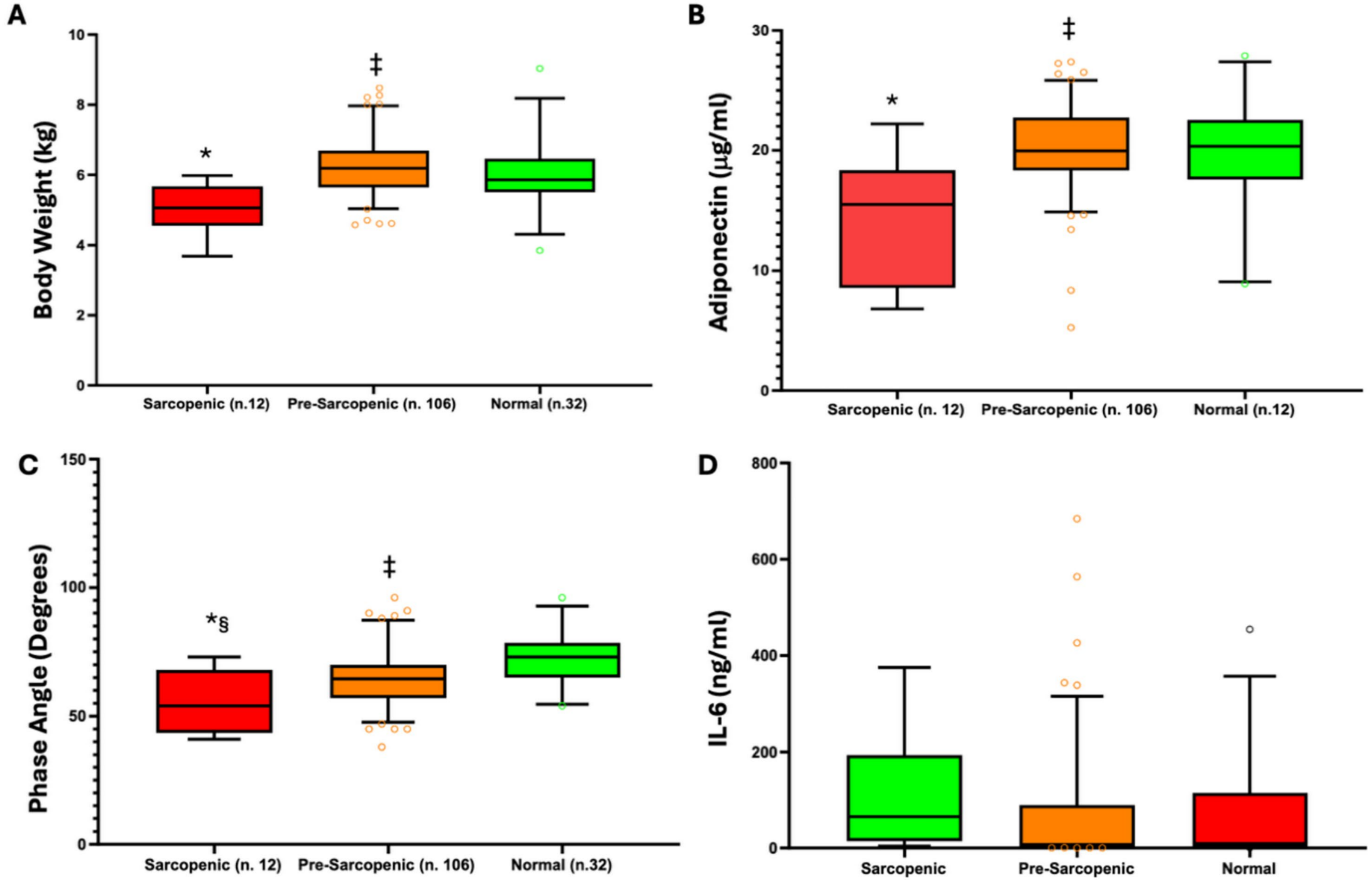
Body weight **(A)**, adiponectin **(B)**, phase angle **(C)**, and IL-6 **(D)** distribution across patients, sub-divided according to the sarcopenic status. *p < 0.001 vs. pre-sarcopenic and non-sarcopenic; ‡ *p* = 0.373 vs. non-sarcopenic.

Figure 1 shows the parameters that best allow to subdivide the CD population according to the sarcopenic state, which are BMI, PhA, adiponectin and IL-6.

### 3.4 HMW oligomers are related to sarcopenia status in CD patients

**Figure 2:**
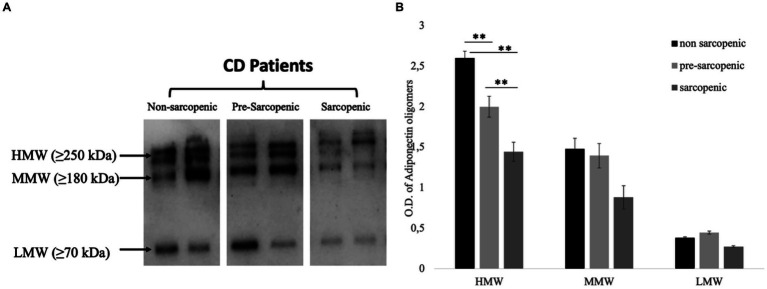
Oligomeric distribution of adiponectin in CD patients. **(A)** Representative western blot images for HMW, MMW, and LMW adiponectin oligomers in the serum of non-sarcopenic, pre-sarcopenic, and sarcopenic patients diagnosed with CD. **(B)** Graphical representation of pixel quantization of analyzed CD patients. *p < 0.001.

Additionally, we analyzed the adiponectin oligomers’ distributions by WB. The results of WB analysis showed three bands corresponding to HMW (≥250 kDa), MMW (≥180 kDa), and LMW (≥70 kDa) in CD patients (Figure 2). Interestingly, the densitometric analysis (Figure 2B) highlights a significant increase in HMW oligomers in the sarcopenic subgroup.

### 3.5 ROC analysis

**Figure 3:**
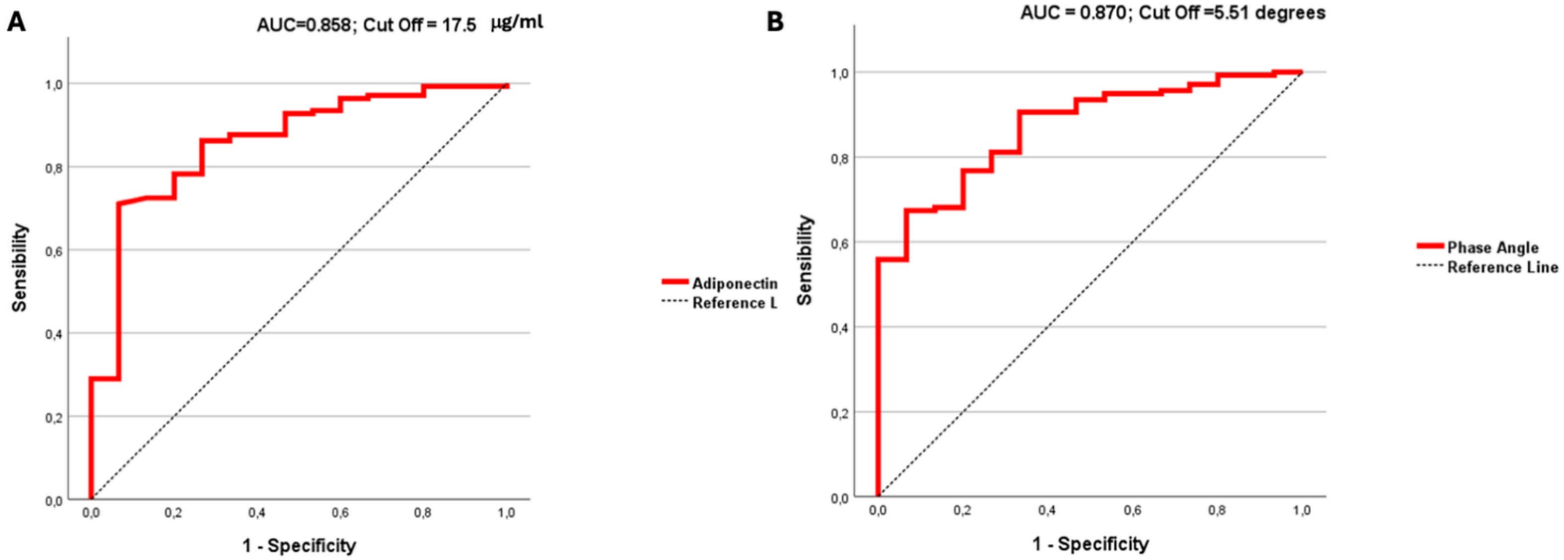
ROC curve analysis shows that serum adiponectin levels panel **(A)** and phase angle panel **(B)** are the parameters best correlated with the risk of sarcopenia in CD population.

To clarify whether and which anthropometric parameters and/or (B) serum levels of inflammatory parameters could be considered a potential predictive indicator in CD patients, we performed a Receiver Operating Characteristic (ROC) curve analysis; the results are shown in Figure 3, panels A and B. In detail, the ROC curves show that serum adiponectin levels and PhA (panel A) (panel B) are the parameters best correlated with the risk of sarcopenia in our population (AUC phase angle: 0.870, AUC adiponectin: 0.858). A cut-off value of 17.5 for adiponectin and 5.5 for phase angle was identified by ROC curve analysis as optimal for discriminating patients with sarcopenia. ROC curves including additional parameters age, gender, age, height, weight, FFM, IL-1, IL-6, TNF-*α*, and lymphocytes are shown in Supplementary Figure 2.

## 4 Discussion

In this study, we showed that both serum adiponectin and PhA are independently associated with sarcopenia in patients with CD. To our knowledge, this is the first study to combine functional, biochemical, and molecular analyses to explore the adipose–muscle interplay in CD-related sarcopenia. In particular, adiponectin and PhA resulted significantly reduced in sarcopenic compared with non-sarcopenic and pre-sarcopenic CD subjects, suggesting their potential role as early biomarkers of muscle deterioration in CD. The observed reduction in adiponectin concentrations, together with the decreased PhA, could reflect the combined effects of systemic inflammation, altered adipose tissue metabolism, and impaired cellular integrity typical of chronic intestinal inflammation.

Sarcopenia is increasingly recognized as a significant extraintestinal manifestation of CD (21) and a significant parameter for risk stratification. It affects up to 50% of patients with Crohn’s disease and contributes to poor clinical outcomes, including fatigue, reduced quality of life, and higher hospitalization rates (6, 22). In our cohort, 8% of patients met the new ESGW criteria for sarcopenia and 71% for pre-sarcopenia, confirming the high prevalence of muscle impairment even in patients with clinically stable CD (23). The reported prevalence of sarcopenia in Crohn’s disease varies widely across studies, ranging from approximately 10 to 50%. In many clinical cohorts, however, the prevalence is around 15–25%. These differences are largely explained by variability in diagnostic criteria, methods used to assess muscle mass (CT, DXA or BIA), and disease activity at the time of evaluation (24–26).

Interestingly, the CD sarcopenic patients within our cohort have a significantly younger mean age and are characterized by lower BMI, lean body mass, and skeletal muscle mass; these observations suggest that the onset of sarcopenia in CD may not be exclusively age-related, but rather due to and exacerbated by disease-related metabolic and inflammatory disorders. Accordingly, metabolic and endocrine changes (insulin resistance, low IGF-1/testosterone in some) have been described to contribute to incidence of sarcopenia in CD (7, 24). Also, chronic systemic inflammation, typical of CD disease, activates proteolytic pathways and causes anabolic resistance which can trigger sarcopenia.

Adiponectin, a key adipokine secreted by adipose tissue, exerts anti-inflammatory, insulin sensitizing, and antioxidant properties. Its role in muscle metabolism has been previously widely highlighted in both metabolic and chronic inflammatory diseases (25). Our findings of more reduced serum adiponectin in sarcopenic CD patients are consistent with the hypothesis that impaired functions of adipose tissue contribute to muscle wasting through the dysregulation of adipokine secretion. In addition, since the values did not substantially differ in pre-sarcopenic patients, our findings may suggest that the observed reduction in adiponectin levels could represent a marker of advanced sarcopenia rather than an early metabolic alteration associated with muscle mass decline. Although total adiponectin is generally regarded as protective, its oligomeric distribution may be crucial in determining biological activity. In general, adiponectin behavior in chronic intestinal disease is heterogeneous. In fact, accordingly to our results, both serum and mesenteric adipose (“creeping fat”) in CD secretes increased adiponectin relative to non-diseased fat (29, 30). Additionally, CD cohorts with active disease, malnutrition have elevated circulating adiponectin vs. controls or vs. quiescent CD patients (31). Whereas broader sarcopenia research finds an association between higher adiponectin and lower muscle mass, to our knowledge, direct longitudinal studies specifically linking serum adiponectin levels to sarcopenia in CD are not present. Our findings expand on earlier reports on adipokine dysregulation in CD. While resistin has been linked to active disease (31), adiponectin appears to act as a protective mediator. *In vitro* studies (32) demonstrated that adiponectin counteracts pro-inflammatory cytokine toxicity through AdipoR1-mediated NF-κB inhibition. The present study corroborates these mechanisms *in vivo*, showing that sarcopenic CD patients exhibit lower circulating adiponectin, particularly in its HMW isoforms, which are recognized as the most biologically active forms involved in insulin sensitization and anti-inflammatory signaling. The HMW complexes represent the most biologically active form and are primarily responsible for activating adiponectin receptor signaling pathways, including AMPK activation in skeletal muscle. Chronic inflammation, which characterizes Crohn’s disease, is associated with elevated levels of pro-inflammatory cytokines such as TNF-*α* and IL-6, which may interfere with adiponectin multimerization and secretion. In addition, inflammatory and oxidative stress conditions can impair the formation and stability of higher-order adiponectin complexes, which require specific post-translational modifications and proper disulfide bond formation in the endoplasmic reticulum. This could preferentially reduce the HMW fraction, leading to decreased adiponectin signaling in skeletal muscle. Such a mechanism may contribute to impaired muscle metabolism and the development of sarcopenia in CD patients.

Reduced PhA values indicate impaired cellular function and nutritional deterioration (33). In our study, CD patients with sarcopenic had significantly lower PhA values, confirming its usefulness as a non-invasive and cost-effective marker of nutritional and muscle status in this disease. Previous studies have reported similar findings in inflammatory bowel disease (IBD), where PhA appears to be correlated with disease activity, inflammatory markers, and muscle mass (34, 35). Furthermore, the ROC analysis confirmed that both adiponectin and PhA are strong predictors of sarcopenia, highlighting their potential as complementary biomarkers for early risk stratification. Interestingly, the strong association between PhA and adiponectin suggests a pathophysiological pathway linking cellular membrane integrity and metabolic resilience. The combined predictive performance of these two markers (AUC = 0.87) highlights their potential utility for early detection of sarcopenia in CD patients. Accordingly, Thibault et al. (36) found that PhA was significantly lower in CD than in controls, especially in active disease and associated with lower muscle mass and higher CRP. Barazzoni et al. (37), Clin Nutr ESPEN, 2018, in 69 CD patients, evidenced a lower PhA in patients with active disease, malnutrition, and sarcopenia with a positive correlation between PhA and muscle mass and handgrip strength. In accordance with our data, they also identified PhA ≤5.4° as predictive of sarcopenia through ROC analysis. Santos et al. (38), Clin Nutr, 2021 found a lower PhA in sarcopenic vs. non-sarcopenic patients and the usefulness of PhA in the prediction of hospitalization risk. The identification of adiponectin and phase angle cut-off values (17.5 and 5.5, respectively) provides clinically relevant thresholds for sarcopenia detection.

Recent evidence suggests that the gut–liver–muscle axis may play a key role in mediating the systemic metabolic and inflammatory alterations observed in CD. Chronic intestinal inflammation is associated with increased circulating levels of pro-inflammatory cytokines, including TNF-*α* and IL-6, which may interfere with adipose tissue signaling and suppress the production of adipokines such as adiponectin. Within this framework, intestinal inflammation could contribute to systemic metabolic dysregulation through coordinated interactions between the gut, liver and skeletal muscle. Although inflammatory cytokines such as IL-6 and TNF-*α* resulted increased in sarcopenic CD subjects, the differences did not reach statistical significance in our cohort, likely due to the limited number of subjects in this subgroup and the relatively stable disease phase of our cohort. Nonetheless, the observed trends are consistent with the established link between chronic inflammation, catabolic signaling, and muscle degradation. Accordingly, a few reports described an increase of both TNF-α and IL-6 in CD (39). Altogether, our data highlight the close interlink between adipose tissue and muscle tissue in Crohn’s disease. Interestingly, dysregulated adipokine secretion, low-grade inflammation, and nutritional deficiencies appear to synergistically correlate to muscle loss.

Some limitations should be evidenced: The cross-sectional design of the study precludes causal inferences, and the relatively small number of sarcopenic patients may have limited the statistical power of some comparisons. Furthermore, physical activity and food intake were not assessed, although both factors influence muscle mass and adipokine metabolism. Nevertheless, the integration of functional and molecular biomarkers provides novel insights into the metabolic crosstalk underlying muscle loss in CD. Lastly, the absence of data from an independent validation cohort.

In conclusion, this study, by demonstrating that reduced adiponectin levels and PhA are closely associated with sarcopenia in Crohn’s disease, supports the concept that metabolic and nutritional disorders play a fundamental role in muscle loss in CD patients. Our study indicates that adiponectin and PhA may serve as complementary biomarkers for an early diagnosis and monitoring of sarcopenia in CD, paving the way for an integrated and multidisciplinary management strategies that address both inflammation and nutritional status. Indeed, the coexistence of chronic inflammation, adipose tissue dysfunction, and sarcopenia appears to worsen the clinical CD phenotype and also highlights the bidirectional crosstalk between muscle and adipose tissue in chronic intestinal inflammation. Understanding the molecular and physiological mechanisms linking adipose tissue to sarcopenia in CD is essential for developing novel integrated therapeutic strategies that aim not only to reduce inflammation but also to maintain metabolic and nutritional homeostasis.

## Data Availability

The raw data supporting the conclusions of this article will be made available by the authors, without undue reservation.

## Footnotes

1
http://rsbweb.nih.gov.ij/
