# Supplementary figures and images for: Adiponectin and phase angle in the assessment of sarcopenia in Crohn’s disease: beyond muscle mass
Source: Front Nutr. 2026 Apr 23;13:1753532. doi: 10.3389/fnut.2026.1753532 (PMC13149123; doi:10.3389/fnut.2026.1753532)

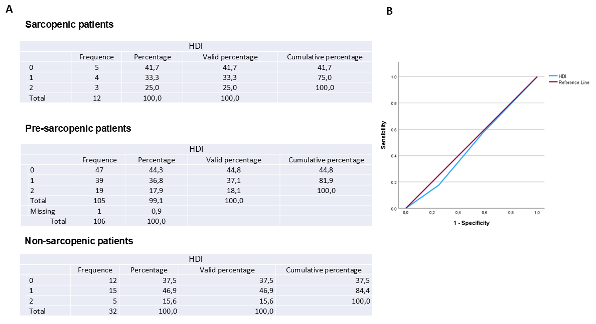

Supplement: Supplementary file 1 [file Image_1.PNG]

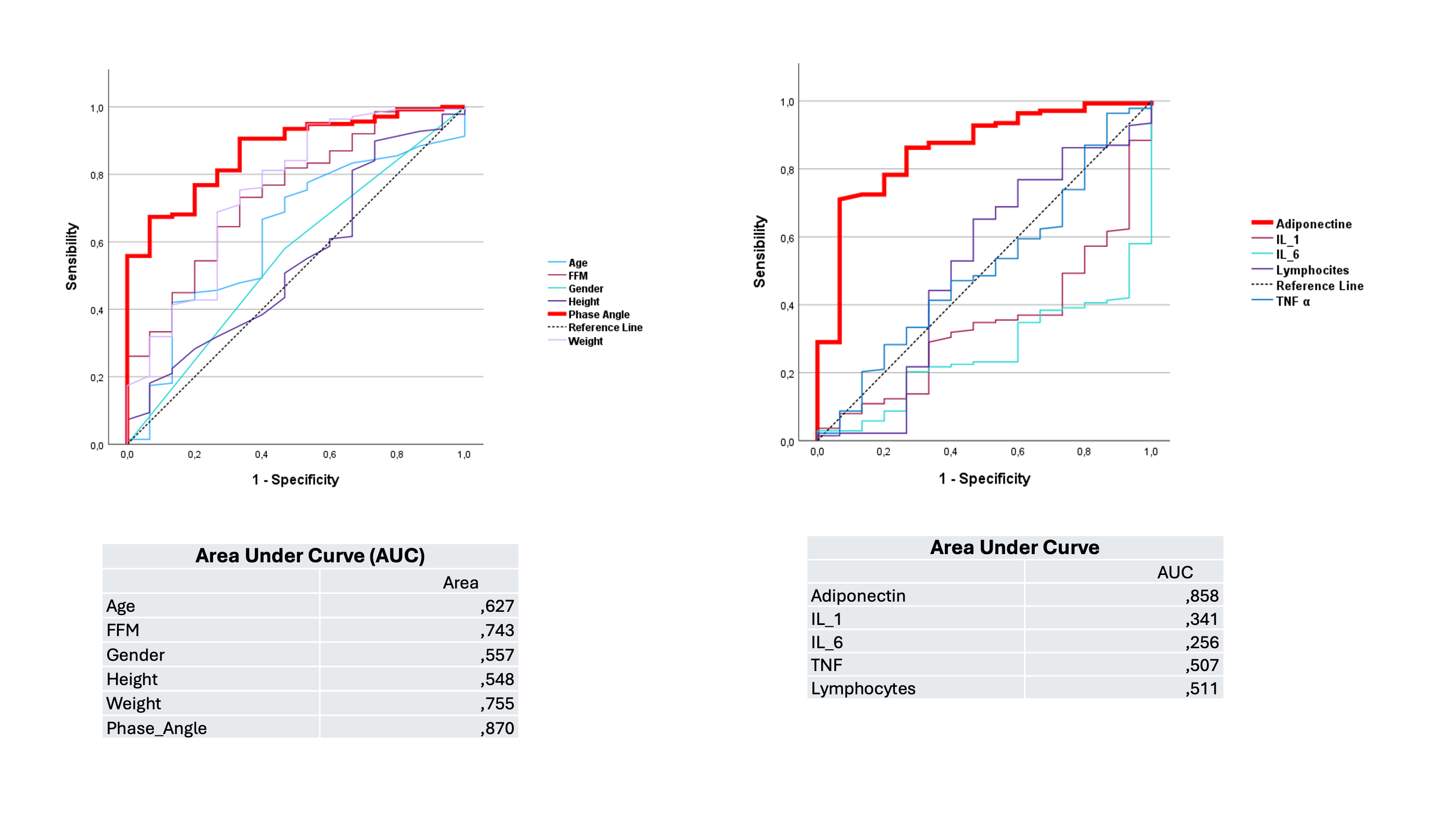

Supplement: Supplementary file 2 [file Image_2.TIFF]
